# Developing a Canadian evaluation framework for patient and public engagement in research: study protocol

**DOI:** 10.1186/s40900-021-00255-4

**Published:** 2021-02-25

**Authors:** Audrey L’Espérance, Nadia O’Brien, Alexandre Grégoire, Julia Abelson, Carolyn Canfield, Claudio Del Grande, Maman Joyce Dogba, Carol Fancott, Mary Anne Levasseur, Christine Loignon, Annette Majnemer, Marie-Pascale Pomey, Jananee Rasiah, Jon Salsberg, Maria Santana, Marie-Claude Tremblay, Robin Urquhart, Antoine Boivin

**Affiliations:** 1 Center of Excellence for Partnership with patients and the public, University of Montreal Hospital Research Center (CRCHUM), Montreal, Canada; 2 Canada Research Chair in Partnership with Patients and the Public, University of Montreal Hospital Research Center, Montreal, Canada; 3 Department of Health Research Methods, Evidence & Impact, McMaster University, Hamilton, Canada; 4 Patient Advisors Network, Montreal, Canada; 5 University of Montreal Hospital Research Center (CRCHUM), Montreal, Canada; 6 Centre de recherche en santé durable – VITAM. Department of family and Emergency Medicine. Université Laval, Quebec, Canada; 7 Canadian Foundation for Healthcare Improvement, Ottawa, Canada; 8 Centre de recherche Charles-Le Moyne – Saguenay – Lac-Saint-Jean sur les innovations en santé (CR-CSIS). Département de médecine de famille, Université de Sherbrooke, Québec, Canada; 9 Research Institute of the McGill University Health Centre. Faculty of Medicine and Health Sciences, McGill University, Montréal, Canada; 10 School of Public Health, Université de Montréal, Montreal, Canada; 11 Alberta SPOR SUPPORT Unit. Faculty of Health Disciplines, Athabasca University, Alberta, Canada; 12 Health Research Institute. University of Limerick School of Medicine, Limerick, Ireland; 13 Departments of Pediatrics and Community Health Sciences, University of Calgary, Calgary, Canada; 14 Department of Community Health and Epidemiology, Dalhousie University, Halifax, Canada; 15 Department of family and emergency medicine, Université de Montréal, Montreal, Canada

**Keywords:** Patient and public engagement, Patient-oriented research, Participatory action-research, Evaluation

## Abstract

**Background:**

Patient and public engagement (PPE) in research is growing internationally, and with it, the interest for its evaluation. In Canada, the Strategy for Patient-Oriented Research has generated national momentum and opportunities for greater PPE in research and health-system transformation. As is the case with most countries, the Canadian research community lacks a common evaluation framework for PPE, thus limiting our capacity to ensure integrity between principles and practices, learn across projects, identify common areas for improvement, and assess the impacts of engagement.

**Objective:**

This project aims to build a national adaptable framework for the evaluation of PPE in research, by:

1. Building consensus on common evaluation criteria and indicators for PPE in research;
2. Defining recommendations to implement and adapt the framework to specific populations.

**Methods:**

Using a collaborative action-research approach, a national coalition of patient-oriented research leaders, (patient and community partners, engagement practitioners, researchers and health system leaders) will co-design the evaluation framework. We will develop core evaluation domains of the logic model by conducting a series of virtual consensus meetings using a nominal group technique with 50 patient partners and engagement practitioners, identified through 18 national research organizations. We will then conduct two Delphi rounds to prioritize process and impact indicators with 200 participants purposely recruited to include respondents from seldom-heard groups. Six expert working groups will define recommendations to implement and adapt the framework to research with specific populations, including Indigenous communities, immigrants, people with intellectual and physical disabilities, caregivers, and people with low literacy. Each step of framework development will be guided by an equity, diversity and inclusion approach in an effort to ensure that the participants engaged, the content produced, and the adaptation strategies proposed are relevant to diverse PPE.

**Discussion:**

The potential contributions of this project are threefold: 1) support a national learning environment for engagement by offering a common blueprint for collaborative evaluation to the Canadian research community; 2) inform the international research community on potential (virtual) methodologies to build national consensus on common engagement evaluation frameworks; and 3) illustrate a shared attempt to engage patients and researchers in a strategic national initiative to strengthen evaluation capacity for PPE.

## Plain English summary

Patients and members of the public increasingly participate as partners in health and health care research. However, evaluation of such initiatives are rare. This limits what we know about how to better engage with people. The goal of this project is to develop a national framework to evaluate patient and public engagement in Canada. We aim to provide local teams with a common blueprint to evaluate engagement activities. We will bring together patients, community partners, researchers, and health system leaders across Canada to create this evaluation framework. We will build consensus using online workshops with voting and prioritizing exercises. At each step in the process, we will seek participants with diverse backgrounds and health care experiences, such as, people from Indigenous communities, immigrants and newcomers, people with intellectual or physical disabilities, and people with lower levels of literacy. Participants will also contribute different perspectives as researchers, patients and caregivers. Knowing that certain groups encounter barriers to participation, patients will help design both methods and content for the research activities. Finally, expert working groups, co-led by researchers with patients and community members, will recommend how to adapt the framework for specific populations and seldom-heard groups. We expect this project to improve patient and public engagement by helping teams to reflect on their engagement initiatives. It is also an example of building consensus for evaluation that could inspire researchers in other countries.

## Introduction

Patient and public engagement (PPE)^1^ in research is growing internationally, and so is interest in its evaluation. Around the world, the health research community increasingly recognizes the need to engage with a wider diversity of patients and communities, in a way that is relevant, meaningful, reciprocal and impactful. There is a need for robust evaluation and stronger evidence regarding PPE in research, from accountability, scientific and continuous improvement perspectives. Several authors (e.g. [2, 6, 14, 28, 29]) describe how the knowledge gap between “what we want to achieve” and “what we know we achieved” can negatively affect those who are skeptical of the benefits of PPE in research (fuelling their reluctance to engage), but can also hinder the work of those who are convinced of its importance (limiting their efforts to improve engagement practices over time). The call for sound evaluation resonates both with those approaching engagement from a normative perspective (seeing engagement as having intrinsic value) or from an instrumental perspective (seeing engagement as a means to an end) [7, 8].

In 2019, a systematic review of patient and public engagement evaluation frameworks was conducted by Greenhalgh and colleagues [15]. It showed how diverse and theoretically heterogeneous this literature is. Their final data set consisted of 65 evaluation frameworks. The authors concluded that the diversity in purposes and scientific underpinning of existing frameworks limited transferability across contexts. They concluded that *“a single, one-size-fits-all framework may be less useful than a range of resources that can be adapted and combined in a locally generated co-design activity”* developed with patient collaborators [15]. Whether and to what extent a diverse set of stakeholders (e.g. patients, researchers, health system leaders) can agree on a common evaluation framework for patient and public engagement, and how such consensus can be achieved, remains an open question.

### Objectives

This article reports on the protocol of a funded study, whose primary goal is to develop a national adaptable framework for the evaluation of PPE in research. Specific objectives are to:

1. Build consensus within the Canadian research community on the core evaluation criteria, process and impact indicators of PPE in research;
2. Define experts’ recommendations on the implementation of the evaluation framework and its adaptation to different populations, with a focus on equity, inclusion and diversity.

The PPE evaluation framework is intended to serve as a common minimal structure around which the Canadian research community will be able to build their own evaluation initiatives; thereby promoting comparability across evaluations, while allowing for flexibility and adaptation to different populations and contexts. Our intention is to offer enough supporting theory and guidance for research organizations and partners to build their own evaluation in a way that: 1) aligns with common engagement principles outlined in the Canadian Strategy for Patient-Oriented Research; 2) is adaptable to specific contexts of engagement (settings, populations, type and domains of research, etc.), and 3) allows for mutual learning and understanding across projects and organizations by defining core evaluation standards applicable across Canada.

### Canadian context

The Canadian Institutes of Health Research’s (CIHR) Strategy for Patient-Oriented Research (SPOR) was launched in 2011 to support “a continuum of research that engages patients as partners, focusses on patient-identified priorities and improves patient outcomes” [10]. SPOR generated significant momentum and opportunities for greater PPE in research and health-system transformation. Nationally, SPOR’s four principles of patient and public engagement – *Inclusiveness, Support*, *Mutual Respect*, and *Co-Building -* have steered practices of PPE and reinforced them through a set of research-focused entities meant to build capacity for, and implement, engagement amongst researchers and their partners. These SPOR funded entities include SUPPORT Units (patient-oriented research methodology support infrastructure, providing practical support for patient and public engagement in research) and SPOR Networks (national research networks focused on specific diseases or topic areas, engaging patients and members of the public in their operations).

While SPOR has fostered the practice of patient and public engagement in Canadian health research, current engagement initiatives are rarely evaluated, or are evaluated with different frameworks. Lack of common evaluation framework limits capacity to:

- Ensure *concordance* between guiding principles and engagement practices;
- *Learn across projects* implemented throughout the country;
- *Monitor progress* and improve practices over time
- Assess the *outcomes and impacts* of engagement (what difference does it make, in which circumstances and for whom?).

Recent developments in the field of PPE evaluation are helping to address this gap. In collaboration with national SPOR partners, members of our research team have published a systematic review of evaluation tools for PPE in research [7, 8]; assembled existing instruments in an open access online evaluation toolkit (https://ceppp.ca/en/our-projects/evaluation-toolkit/); and developed original, validated evaluation instruments (e.g. [3, 4]). While these endeavours provide the Canadian research community with a set of individual evaluation instruments, we still lack a common evaluation framework to serve as a blueprint for building collaborative evaluation projects or comparing engagement practices. Existing evaluation frameworks tend to be limited to single engagement interventions, are difficult to transfer across research projects, and/or have limited applicability to the Canadian research context. As a result, published evaluations are characterized by a number of small studies that limit our ability to build a common knowledge base and sustain a learning environment that builds best practices for PPE.

A national initiative like SPOR provides an opportunity to develop consensus around evaluation. The level of coordination and collaboration between SPOR entities (Units and Networks) with respect to PPE could provide sufficient common ground to adopt a common framework, which could then be adapted to specific local contexts and populations.

## Methods

### Research approach

Using a collaborative action-research approach [22], a national coalition of patient-oriented research leaders, including SPOR SUPPORT Units and Research Networks, patient and community partners, engagement experts, and health system leaders, will collaborate to co-design this evaluation framework. Hence, as much as this project will build on the existing international science of PPE evaluation, the consensus building endeavour will ensure that PPE evaluation is tailored to the national context and will consolidate ongoing collaboration amongst the PPE research community in Canada.

**Fig. 1:**
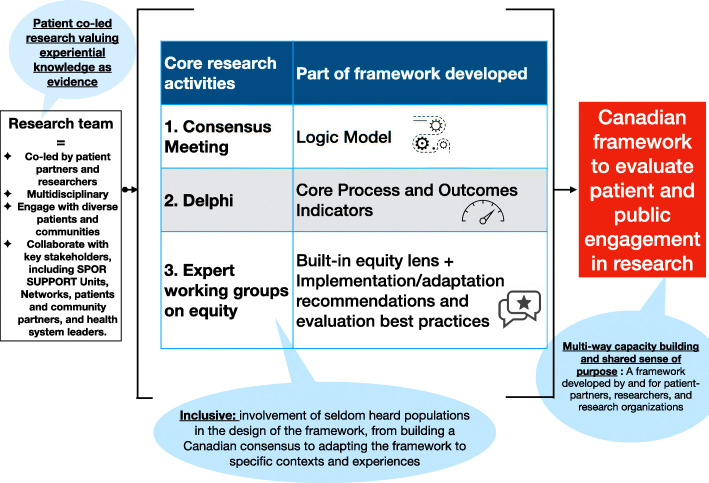
Project overview

The main research phases are depicted in Fig. 1, including: 1) development of a logic model (via consensus meetings), 2) agreement on a core set of process and outcome indicators (via a Delphi process) and 3) development of implementation guidelines (via co-led expert working groups on implementation and adaptation to seldom-heard populations).

Our evaluation framework development process will be supported by international evidence based on systematic reviews of evaluation frameworks for patient and public engagement in research [9, 11, 12, 15, 18], a systematic review of existing evaluation instruments [8], evaluation principles for evaluating patient and public engagement in research [8], and a systematic review of patient and public engagement outcome indicators and evaluation methods [32]. This preparation work will be used to produce background evidence documents for expert working group members, to develop Delphi questionnaires and prepare consensus meeting participants.

With this general understanding of what PPE in research looks like and what the literature broadly considers as successful PPE processes and impacts as a base (e.g. [16, 17]), this project will take the next steps to define what success in PPE looks like for the Canadian research community in particular. Consensus-generating participatory methodologies will be used (Nominal Group Technique and Delphi Method) to consolidate a logic model and build consensus on a core set of PPE evaluation standards. These steps will help us answer the following questions:

1. What is meaningful PPE in research for the Canadian research community? (Process indicators);
2. What does successful engagement look like for stakeholders of the SPOR? (Outcome indicators); and
3. How should we approach the evaluation of PPE engagement in research? (Evaluation criteria)

As Rowe and Frewer [26] described, defining the term *effectiveness/success/meaningfulness* is paramount to sound evaluation. Unless there is a clear definition of what it means for a PPE initiative to be *effective/successful/meaningful* in a given context, it is not possible to develop measures and methods that enable the evaluation of patient and public engagement. However, not universal in nature, the definition of what represents success in a given context needs to be adopted by the key potential users of the evaluation framework. For this reason, our project will work collaboratively with engagement leads, patient partners and researchers from the Canadian SPOR community specifically to define the parameters of these terms.

We recognize that different evaluation perspectives may exist amongst interested parties. Although competing evaluation purposes may coexist, we assume that key partners in patient-oriented research are more likely to measure their engagement activities against a common set of goals if they set those goals together, as a community. Previous studies have documented research partners’ interest in documenting whether their collaborative efforts make a difference in the research process (see [1]). Our research team will build on the ongoing Canadian collaborations around PPE within SPOR to bring about such consensus and to create a common understanding around what a SPOR Patient Engagement Evaluation Framework should look like.

#### Virtual consensus meeting

The first consensus-building exercise, employing the nominal group technique, will consist of a structured facilitation strategy for group brainstorming that will allow for a larger conversation to occur between research stakeholders, including patient partners, engagement experts, and researchers. This will fulfill three purposes: 1) building research community mobilization around the construction of the framework; 2) fostering common understanding of PPE evaluation, and 3) generating ideas about core evaluation domains of the logic model (i.e. aligning objectives of PPE in research with evaluation criteria; PPE process dimensions with categories of process indicators; PPE outputs dimensions with categories of outcome indicators). The nominal group technique is a structured variation of small group discussion methods [31]. This structured process aims at preventing discussions from being dominated by a single person or group, by encouraging less vocal group members to participate in the prioritization of solutions and recommendations.

Collaboration with 18 national SPOR Networks, SUPPORT Units and research organizations (from all Canadian provinces and covering research focus in primary care, diabetes, chronic pain, mental health, developmental disability, chronic diseases, kidney diseases) will provide broad representation from the Canadian patient-oriented research community, mobilizing participants with experience on PPE in research and its evaluation. Our consensus panel for the nominal group technique will involve approximately 50 individuals identified through partner organizations (Acknowledgement section). All SPOR funded-entities will be invited to nominate two or three participants – one patient or community partner and one engagement practitioner (eg. research professional, engagement facilitator or researcher with engagement experience), with a focus on identifying participants with engagement expertise and a diversity of perspectives. In addition, the meeting will include other specialists in the field of PPE, including representatives from the Patient Advisors Network, a patient-led community of practice bringing together experienced patient partners in research, and the Canadian Foundation for Healthcare Improvement, a pan-Canadian organisation dedicated to fostering research and healthcare improvement with expertise in patient and public engagement [13].

This event, that will consist of four virtual meetings held at different dates, was originally intended to be held in person. However, the advent of COVID-19 required us to re-evaluate our original strategy. Within the COVID-19 context, we will hold this consensus process online, in accordance with best practice recommendations for virtual consensus-building (e.g. [24, 30]). The consensus meeting will still include the 8 key phases of a nominal technical group, but it will occur in sequence over 4 virtual meetings, each of which will be carried out with about 12 to 15 participants to facilitate group discussion and sharing. Sub-groups will run in parallel (meetings #1-#2-#3), with summaries from discussions shared among all participants at the plenary discussion phase (meeting #4), as described below.

##### Meeting 1- preparation phase

A preparation webinar will be offered to participants. This webinar will provide a quick overview of what is currently known about evaluating PPE in research. This will allow participants to have a common knowledge and understanding of the concepts being discussed, and to clarify the project goal and set the stage for discussion rounds. The webinar will address what an evaluation criterion is and what evaluation dimensions are, providing specific examples to the participants. The webinar will be given live with the possibility of a questions and answers period at the end. It will also be recorded in order for participants who won’t be able to attend to watch it asynchronously. Complementary documents will also be available to participants to help them prepare for the following discussions: an outline of project objectives and timeline, a jargon buster, and a synthesis of key articles about PPE evaluation. Ongoing communication with the team is key during the Virtual Consensus Meeting, hence a Slack channel (www.slack.com) will be created and facilitated by a project team member for the duration of Phase 1 to answer questions and orient participants.

##### Meeting 2- ideas generation phase

Virtual Zoom meetings of 120 min will be conducted with each sub-group to generate ideas on evaluation criteria, process and impact indicators. To identify evaluation criteria, participants will be asked to define principles of PPE in research, to describe their perspectives on what makes PPE in research successful. To identify process dimensions, participants will be asked about standards to determine whether a process of PPE in research is going as planned, and about factors that enable meaningful engagement. To identify impact dimensions, participants will discuss the changes they feel should be brought about when patients are engaged in research and on whom or what these change should be observed. Participants will be asked to identify standards that help determine whether a research project involving patients has achieved the desired outcomes.

Research team members will facilitate these meetings on several dates to accommodate a maximum number of participants. We will create sub-groups composed of equal number of professionals and patient partners. Other options will be made available to participants to ease participation in the discussions. They will have the opportunity, after each meetings, to send their ideas and comments in: 1) writing (format chosen by the participant - email, word, etc.) and 2) audio (with recordings sent to the project team and transcribed by a team member).

##### Round Robin phase

Verbatim transcripts of participants’ dialogue (recordings of zoom meetings, transcripts and written statements) will be analyzed with the help of Nvivo to identify specific sets of ideas, themes, and remaining questions/needs for clarification. A summary report will be produced by the project team and circulated amongst participants to help start the next facilitated round.

##### Meeting 3 - clarification phase

At meeting 3, each idea/set of ideas/themes will be discussed one at a time in order to clarify them and/or explain disagreement with emerging positions. These will be 120-min virtual Zoom meetings within sub-groups. Research team members will facilitate the meetings, with several dates proposed to accommodate a maximum number of people. We will create sub-groups composed of equal number of professionals and patient partners.

##### Voting phase

At the end of the *clarification* phase, participants will begin to narrow the list of potential ideas. Building on the clarification phase, each member will rank the ideas they consider from most to least important. Zoom provides a survey application built into the plate-form that we will use to ask for a virtual simultaneous vote.

##### Consolidation phase

The top ideas from each subgroup will be consolidated by the research team, combining any duplicate ideas, and a master list of ideas will be generated for the plenary discussion and final vote. This list will be circulated amongst participants prior to the last closing meeting.

##### Meeting 4 - plenary discussion

A 120-min zoom meeting (one date - set in advance to maximize participation) will be held. Briefly, the facilitator will present to participants each idea for each question and their previous ratings in order to examine inconsistent voting patterns and provide an opportunity for a discussion around these outlier ideas. Ideas with strong support will be maintained whereas those with weak support will be eliminated. Participants will be able to use both the microphone and the chat box to participate in the discussion.

##### Final vote

A final vote will be called at the end of the meeting on the three sets of ideas (criteria, process dimensions, impact dimensions), asking participants to rank ideas generated by the group.

Meeting 2 and 3 will involve the same sub-groups of participants to increase their sense of belonging and ease discussion. We will try as much as possible to recreate online the same dynamics the face-to-face setting would have provided. For each step of the virtual consensus meetings, a facilitator and note taker from the project team will be facilitating the discussion. The meetings will be recorded to ease note taking and maximize content analysis. Using the notes and transcriptions of the meetings, we will proceed to a thematic content analysis using Nvivo. Analysis will focus on: 1) identifying emerging evaluation criteria, processes and indicators (to generate items for the Delphi priority-setting exercise); 2) participants’ interpretation of the meaning of those criteria and indicators (to inform narrative description of indicators and criteria); and 3) areas of consensus and dissent regarding the relative importance of emergent indicators (to inform our analysis of whose voices were dominant or not in generating potential indicators and criteria).

#### Delphi

While the virtual consensus meeting (above) will be used to generate potential evaluation criteria and dimensions, a modified Delphi process will seek to identify those criteria and dimensions that are most important for the community of patient-oriented research experts. The Delphi Technique is a well-known method for structuring (organizing and facilitating) a group communication and consensus process. It is characterized by iterative rounds of questionnaires with systematic feedback between each round. The method allows for large pools of individuals to be involved asynchronously at times and locations convenient to them, while preserving their anonymity. It also allows identification of areas of convergences and dissension among stakeholder groups. For that purpose, two rounds of online questionnaires will seek to determine which standards (process and impact indicators) are most important for the SPOR community. This activity will provide a minimal evaluation structure to be implemented throughout the SPOR environment.

To increase the variety of expertise engaged, the participants invited to take part in the Delphi will cover a broad representation of the Canadian community of patient partners in research and researchers supported/involved within the SPOR environment. We will aim to solicit 200 participants and will be helped by SPOR entities to reach out to their respective members and collaborators to identify potential Delphi participants. SPOR entities will be asked to identify 5 researchers who conduct patient-oriented research in partnership with patients as well as 5 patient partners involved in research. Co-leads of our equity, diversity and inclusion working groups (described below) will also be solicited to identify prospective participants from seldom-heard populations. Purposeful selection of participants will be carried by the research team to ensure that a diversity of perspectives are represented, both from a sociodemographic point of view and in terms of patient experience pathway.

In Round One, we will share our interim report from the consensus building exercise. Panelists will then be asked to prioritize indicators for each dimension, among the list identified during the nominal group technique exercise. Specific prioritization techniques (rating, ranking, choice of scales) will be determined by the research team, based on the number of items emerging from the consensus meetings and pilot testing of the Delphi survey. Participants will be invited to provide optional qualitative justifications for their choices.

In Round Two, participants will receive a summary of Round One results, and will be asked to provide their final, informed priorities, using the same evaluation techniques as used in Round One. These questions are designed to establish the indicators that are the highest priority for the SPOR community. In addition to identifying and ranking indicators, the respondents will also provide input on the chosen wording to identify PPE evaluation standards. This will provide the research team with information to complete common parts of the PPE evaluation framework.

Turnaround time between questionnaires will be 6 weeks. Only two rounds of questionnaires will be sent to participants, as the initial round will already be informed by the results of the consensus meeting. This should increase participation and limit the attrition rate over the process.

We will analyze results for all participants, and by sub-groups (eg. patient partners vs. researchers, sociodemographic characteristics) to identify areas of consensus and dissent among all participants and for distinct groups. These stratified analyses will inform discussions of expert working groups on adaptation of the evaluation framework for specific populations (below).

#### Expert working-groups on implementation and adaptation

While prior phases of this project will focus on general PPE process and outcomes indicators, this approach does not address the reality that many will face in determining the relative importance to assign to each of these elements in the evaluation of PPE initiatives for specific populations. We know that approaches taken to evaluating PPE differ conceptually and methodologically in response to the different populations engaged as well as the purpose of patient and public engagement in a given context. Abelson and Gauvin [1] raised these crucial questions: “*how evaluation criteria or ‘elements of success’ should be weighted in evaluation, by whom, and whether some criteria are more important than others in terms of their contribution to the evaluation”. (p. 7).*

Therefore, in order to answer these questions, six working groups of experts – each co-led by one researcher and one patient/public partner – will work with their respective research and patient/public partner communities in defining recommendations for the implementation and adaptation of the framework. With the results and analysis of the consensus exercises in hand, co-applicants (scientific and patient experts) and collaborators (SPOR Networks and SUPPORT Units) will be involved in designing implementation strategies and outlining fundamental elements of an evaluation design, namely 1) stakeholders’ involvement in the evaluation process, 2) specific evaluation question(s), 3) recommended approaches to evaluation; 4) preferred wording of terms used and formulation of criteria. The six expert working groups will focus on:

1. General recommendations for implementation of the evaluation framework
2. Evaluation adaptation with Indigenous communities
3. Evaluation adaptation with caregivers
4. Evaluation adaptation with immigrant populations
5. Evaluation adaptation for patients with physical and/or intellectual challenges/disabilities
6. Evaluation adaptation for patients with low literacy levels

These six working groups are by no means extensive, other pertinent groups may have included participation from other hardly heard communities as defined by religion, language, gender identity and expression, sexual orientation, age, socio-economic status, and from stigmatized groups such as people using drugs, people living with HIV, or people experiencing homelessness. Our work may thus serve as a template to future teams to adapt the framework to their particular research focus.

The six working groups mobilized within our research will discuss the experience of their own community of patient/public partners in order to identify the contexts and processes that could likely be obstacles or facilitators to contributing engagement practices, and thus outline how to adapt the PPE evaluation framework for these populations. Discussions around the adaptation and implementation of the general evaluation framework for PPE will focus on evaluation indicators and standards that should be emphasized, added or removed, when engaging with specific populations. Those discussions will be informed by population-specific analysis of responses within the Consensus Building Exercise and Delphi process.

Overall, the consensus meetings will have led to the construction of the logic model, the Delphi will have set the SPOR community’s choices for priority process and outcome indicators, and the expert working-groups will provide recommendations on adaptation and implementation of the national framework to specific populations.

### Equity, diversity and inclusion

There is strong evidence from the literature that some patient groups face more barriers than others to engage in research [25, 27]. For instance, we know that women are overrepresented in the patient communities and more so in some health research areas than others (e.g. [20, 21]). Conversely, people from ethnic minority groups, living with poverty, or with intellectual disability face specific barriers to engagement that may hamper their meaningful involvement in research [19].

Our equity, diversity and inclusion strategy has three complementary goals:

1. That *participants* in the development of the national evaluation framework include a diversity of perspectives and is inclusive of seldom-heard populations;
2. That the *content* of the national evaluation framework addresses evaluation dimensions that are informed by equity, diversity and inclusion considerations;
3. That *implementation* of the national evaluation framework is adapted to specific needs and perspectives of seldom-heard populations.

Therefore, from the start of the project, the research team will make sure equity is intertwined in each stage of its design and development. This will involve co-designing each phase of research with patients from seldom heard populations and experts in the field. Our research team actively recruited experts on equity in PPE to foster an inclusive design process. Although inclusion of all perspectives is not feasible, we will pay special attention to 5 seldom-heard groups of particular importance for PPE in research within the Canadian context: Indigenous communities, new immigrants, people with intellectual and physical disability, people with low literacy levels, and caregivers.

At phase 1 – Consensus Meeting – a self-identification questionnaire will help to characterize the sociodemographic profiles of consensus-building participants. At this stage of the process, the focus is on the involvement of participants with expertise and experience in PPE in research, as identified by SPOR research entities. We expect this recruitment strategy will reflect the fact that certain socio-demographic groups are over or under-represented. By highlighting these profiles, it will be easier to take note of potential exclusions and to purposely recruit participants for phases 2 and 3 of the project. We will also have the opportunity to code the transcripts of the meetings in order to analyze whether certain issues of diversity, equity and inclusion emerged, by whom they were raised and whether these elements were reflected in the dimensions and criteria chosen to constitute the logic model.

At phase 2 – Delphi – the research team will recruit purposefully to ensure that a diversity of perspectives are represented, both from a sociodemographic point of view and in terms of patient experience pathway. This includes recruitment of participants from identified seldom heard groups. We will work to adapt the Delphi questionnaire in collaboration with representatives of the five seldom-heard communities to see what kind of adaptations will need to be made to ease participation as much as possible. Among other things, we could consider to roll-out the questionnaire in multiple ways, including by telephone, to help include participants with lower literacy or disabilities. A series of identification questions will help us clarify the profile of Delphi participants in order to better understand the differences and similarities between specific seldom-heard populations preferences and the general group. In addition to allowing the expert working groups to prepare and guide their work, this information will help us to better distinguish between areas of consensus (core evaluation framework) and areas of dissent (informing guidelines for implementation and adaptation).

At phase 3 – Experts-working groups – five of six expert working groups will analyze the simultaneous interactions between different social categories that make up a patient’s social identity, as well as the impact of power imbalances on the involvement of individuals who often carry the burden of illness. Using the list of questions defined by Shimmin et al. [27], these working groups will discuss the experience of different communities of patient partners in research and answer three questions with the angle of better understanding the specificities of embarking in PPE evaluation with these populations:

1. Are the evaluation criteria and dimensions representative of their experience? If yes, why? If no, what would be more relevant?
2. What evaluation criteria and dimensions should be emphasized, added or removed when evaluating PPE in conducting research with your specific group?
3. Are there evaluation approaches that need to be adopted and protocols implemented in order to adequately embark in the evaluation of PPE?

This project is supported by a research team composed of experts who have been working with these five communities for several years and who have built trusting relationships with participants. The working groups will all require a specific strategy to enhance equity and inclusion. Thus, the first step will be to determine the terms of the collaboration and to define the best ways of working together. We do not want to pre-determine this strategy at the protocol stage. Co-construction is key and will require us to allocate it time and plan this phase of the project accordingly.

Adding to the aforementioned analysis, sex- and gender-based analysis will be led by the research team at the 3 phases to better understand the gendered nature of PPE in Canada and how these factors can affect the process and outcomes of PPE. This health equity exploration will allow for a more inclusive and reflective approach to PPE evaluation and ease the adaptation and implementation of the common framework to specific population needs.

### Patient and public engagement

The overarching goal of our engagement strategy within the project is to enhance the relevance, value and usefulness of the proposed evaluation framework to help patients and researchers reflect together on their research partnership. Patient and public partners are involved at every level (strategic, tactic and operational) and phases (research preparation, execution, and knowledge translation) of this research project. Research agenda-setting and framing of the initial project was conducted with the SPOR national patient and public engagement working group, which includes patient and public partners from across the country with extensive experience in supporting engagement in research. Patient partners with extensive experience in research have been engaged since the onset as co-principal investigators (AG) and co-investigators (CC) on the research team and contributed to study design, protocol development and funding application; they will also contribute to ongoing development of study instruments (eg. consensus meeting questions, Delphi survey), recruitment strategies, data interpretation and knowledge translation. Patient and public partners from the 18 national SPOR Networks, SUPPORT Units and research organizations collaborating on this project will be involved in consensus meetings to design the initial evaluation framework template and logic model, alongside 2 national patient leaders from the Patient Advisors Network, an independant community of people who have received health services or cared for those who are, committed to improving healthcare as advisors across Canada. These patient and public collaborators will further be engaged in the recruitment of participants in the Delphi survey, as well as in the dissemination and implementation of the framework within their organizations and communities. Finally, patient and public partners will act as co-leads of the working groups on equity, inclusion and diversity (described above), contributing to its design, structure, recruitment, facilitation and approval of recommandations. Evaluation of our patient and public engagement dynamics (team interaction, support, contribution, feeling valued, convenience, research environment, procedural requirements and benefits) will be conducted at the end of every stage of the project, using the Patient Engagement in Research Scale [16, 17] with additional questions on the experiences of online engagement, in order to support reflexivity and improvement of our own engagement practices within the project.

### Research in time of COVID-19

Current health challenges around the world have asked us to reconsider the timeline of this project to allow for the logistical adaptations necessary to comply with public health guidelines while maintaining methodological rigor. At the time of publication of this protocol, the first phase of the project will be well underway and consensus meetings should be completed and the transcripts being analyzed. The transition from a face-to-face to a virtual setting will have lengthened this phase of the project from what was originally planned, delaying the Delphi phase by approximately 3 months. The deployment of Delphi is planned for the spring of 2021 for the first round and the summer of 2021 for the second round. Its preparation (recruitment strategy and logistics, questionnaire development, participant recruitment, etc.) is therefore planned for the winter of 2021. As for the expert working groups, all six of them ask for a specific strategy to strengthen and promote the participation and inclusion of the communities involved. In light of this, this last phase will take place between the months of May and December 2021. This 6-month period should give us enough time to adapt the form and pace of the work to the needs of each of these groups of participants. The complete project was originally planned for a one-year period; the COVID-19 pandemic therefore required us to re-plan for an 18-month period.

## Discussion

The project’s potential contributions are threefold: 1) support capacity building for evaluation of PPE within the Canadian research community; 2) inform the international research community on potential virtual methodologies to build national consensus on common evaluation frameworks; and 3) illustrate a shared attempt to engage patients and researchers in a strategic national initiative to strengthen evaluation of PPE.

As early as 1993, Butterfloss et al. called for a systemization and better understanding of what characteristics of collaborative endeavours lead to producing short and long-term impacts on the communities they serve. More recently, Barber et al. [5] demonstrated the benefits of a structured evaluation towards providing evidence on the influence of patient and public engagement on researchers, research processes and research products. For research funders such as CIHR and patient and public engagement capacity building organizations such as SPOR SUPPORT Units and Networks, this project will provide tools to improve monitoring activities, better support PPE in research and offer guidance to improve PPE activities. For patients and members of the public, this project will offer them mechanisms to foster collaboration and feedback loops, contributing simultaneously to patients’ empowerment and competence building [23]. This common evaluation framework will facilitate collaboration, cross-learning and strengthen PPE in research across Canada, while allowing for local adaptations to contexts, populations and engagement approaches. It will help to catalyze best practices and common strategies for PPE implementation and evaluation across the Canadian research community, all the while encouraging continuous reflection on the actions needed to build learning health systems that truly consider and reflect the needs, priorities and perspectives of patients, communities and citizens.

Since transferability across contexts of PPE evaluation frameworks is difficult and potentially inappropriate, the methods used to generate national consensus (the how) may be more transferable than the evaluation frameworks themselves (the results). We share our “how to” protocol hoping to inspire other groups seeking to develop their own PPE evaluation framework with patients and research partners. Co-designing evaluation frameworks and measures could support common understanding of teams regarding the evaluation of patient and public engagement, increase their ability to engage in evaluation, and foster the learning potential resulting from the evaluation process.

This protocol has been co-constructed in partnership with patients who have experience working in health research. In the past years, the evolution of the Canadian research community, their increasing capacities to engage patients in more meaningful ways and the increased number of patient partners active and dedicated to improving research all helped conceive a protocol that reflects the complexity and the maturity of the Canadian patient-oriented research community. This project consists in a multi-phase process that, while anchored in the growing science of PPE, stems from the experiences of the researchers and patient partners that make up our community. This protocol therefore illustrates a shared attempt from patients and researchers to move forward in building common engagement evaluation criteria and standards.

The research team benefits from an unparalleled level of expertise and networking within the SPOR community. All members of the research team maintain privileged relationships with the SPOR Units and Networks, if not themselves leaders in them. In addition, we are building this project upon a 4-year collaboration between PPE leaders across the country that stemmed from a previous Evaluation Toolkit Project. These collaborations will accelerate the coordination of consensus activities. The SPOR Units and Networks will also be our crucial relays to disseminate the Delphi questionnaires throughout the Canadian patient-oriented research community.

Our proposed approach has several limitations. For example, we are aware of the constraints imposed by the COVID-19 pandemic. For the consensus exercise, which was previously designed to take place in person, the transfer in virtual mode will pose a challenge in building a relationship with participants through a video conferencing platform, a barrier that could be amplified for people with low digital literacy or limited access to technologies. In adapting to an online mode for the consensus building exercise, we will adopt three additional mechanisms to facilitate participation: 1) we will hire a research assistant to further support participants and answer clarifying questions by phone or email; 2) host an orientation meeting previous to the actual consensus building exercise; and 3) set up an informal chat line (e.g. Slack) to allow participants to exchange informally, and allow for another mode of communication with the research team. This limitation is a hypothesis rather than an assertion, as only experience will allow us to judge the impact of this transition from face-to-face to virtual consensus-building (e.g. it may decrease barriers to participation for geographical and transportation concerns, and those with caregiver responsibilities). There may even be positive aspects, such as increased participant availability, home comfort, and reflection time between meetings. We will therefore make sure to reflect on the experience of the participants, especially the patient partners, at the end of each phase of the project in order to learn from the process.

Furthermore, although explicit consideration of equity, diversity and inclusion (and its integration in all phases of the project) is a potential strength, we are also conscious that some groups may nonetheless be excluded because of systemic barriers to participation, including recruitment of people with existing engagement experience through research networks, complexity of the concepts being discussed, power dynamics within group processes and choice of a Delphi process to generate consensus based on majorities’ vote. To mitigate these potential limitations, we will: 1) work with seldom-heard groups and experts in engagement equity to inform and analyse the framework development process; 2) document whose voices may be under-represented and lost through voting, by using sub-group analysis; and 3) complement the national framework with a set of recommendations for adaptation to specific seldom-heard populations. Our process for deliberately engaging with target groups known to experience barriers for engagement (e.g. immigrants and Indigenous communities) within the current project could furthermore serve as a template for complementary projects with other communities.

## Conclusion

The science of PPE is evolving rapidly, yet it remains at an early stage and is challenging to apply within the Canadian research landscape. Concrete ways to guide the production of robust, rigorous and systematic evaluation on PPE in research is at the core of this project. Building a national yet adaptable evaluation framework is an essential precondition for comparable PPE evaluation projects, a core missing piece for PPE in Canada. Built-in adaptation to specific populations will allow for more equity and diversity, while tailoring an adaptation process that could be applied to other populations in future versions of the framework. Apart from contributing to the emerging international science of PPE in research, this project will help national practitioners of PPE (organizations, engagement leads, researchers, patients) to conduct rigorous PPE evaluation, share experiences, learn from engagement initiatives, and report on PPE activities and outcomes in a systematic manner.

While this approach has a Canadian focus, the process of developing the evaluation framework could inform similar consensus-building processes in other national and international settings. We see here an opportunity nested at the heart of the science of PPE to share our research and evaluation methods as much as our results. This will allow both a better understanding of the PPE evaluation products (tools or frames) and a greater ability for PPE specialists to adapt them or craft their own. PPE in research is multifaceted and, according to us, it would be threatening to the very nature of PPE to make it a “one-size-fits-all” model. However, the right approach to building an evaluation strategy will ensure that each initiative keeps it’s uniqueness while providing the PPE community with methods to more systematically and globally understand and learn from experiences. This protocol is therefore our proposal for such an approach, intended to be common and adaptable, and to result in a national minimal structure for the evaluation of PPE in research.

## Data Availability

Not applicable.

## Abbreviations

CIHR: Canadian Institutes of Health Research
COVID-19: Severe Acute Respiratory Syndrome-CoV-2 coronavirus
PPE: Patient and public engagement
SPOR: Strategy for Patient-Oriented Research

## Footnotes

1 For this project we adopt the Canadian Institute of Health Research (2019) definition of patient engagement: “Patient engagement in research is an approach that involves meaningful and active collaboration in governance, priority setting, conducting research and knowledge translation. Depending on the context, patient engagement may also engage people who bring the collective voice of specific, affected communities.”
